# Serotype Occurrence, Virulence Profiles, Antimicrobial Resistance and Molecular Characterization of *Salmonella* Isolated from Hospitalized Patients with Gastroenteritis in Great Tunisia between 2010 and 2020

**DOI:** 10.3390/antibiotics12030526

**Published:** 2023-03-06

**Authors:** Walid Oueslati, Mohamed Ridha Rjeibi, Hayet Benyedem, Mounir Jebali, Fatma Souissi, Rachid Selmi, Mohamed Sélim El Asli, Farouk Barguellil, Abdelfettah Ettriqui

**Affiliations:** 1 Laboratory of Management of Animal Production’s Health and Quality, National School of Veterinary Medicine of Sidi Thabet, University Manouba, La Manouba 2010, Tunisia; 2 Departments of Animal Production, National Agronomic Institute, University Carthage, Carthage 1054, Tunisia; 3 Laboratory of Parasitology, National School of Veterinary Medicine of Sidi Thabet, University Manouba, La Manouba 2010, Tunisia; 4 Laboratory of Parasitology, Veterinary Research Institute, University of Tunis El Manar, Tunis 1068, Tunisia; 5 Laboratory of Microorganisms and Environment, Molecular Diagnostic Tools and Emerging and Re-Emerging Infections, Military Hospital of Tunis, University of Tunis El Manar, Tunis 1068, Tunisia; 6 Laboratory of Microbiology, National School of Veterinary Medicine of Sidi Thabet, University Manouba, La Manouba 2010, Tunisia; 7 Laboratory of Bacteriology, Military Hospital of Tunis, Tunis 1008, Tunisia

**Keywords:** *Salmonella*, gastroenteritis, serotypes, virulence, MDR, Tunisia

## Abstract

Non-typhoid *Salmonella* is one of the major causes of food-borne infections worldwide. The aim of the current study is to determine the serotype occurrence, virulence factors and antimicrobial resistance patterns of *Salmonella* isolated from hospitalized patients. The identification of *Salmonella* strains was performed according to REMIC, 2018. The susceptibility of *Salmonella* isolates was assessed against 20 antimicrobials using the disk diffusion method. Some virulence and antimicrobial resistance genes were identified using PCR. Among the 61 isolated *Salmonella* strains, seven serotypes were identified and all were positive for the virulence genes *invA*, *mgtC* and *sirA*. Critical resistance rates (>40%) were detected for tetracycline, nalidixic acid, amoxicillin and fluoroquinolones. However, resistances to ertapenem, ceftazidim, aztreonam and colistin were null. In addition, 33% of the isolated strains were multidrug-resistant (MDR). Moreover, 80% and 60% of *S*. Kentucky isolates were identified as fluoroquinolone-resistant and MDR strains, respectively. The *qnrB* gene was amplified in 63.2% of fluoroquinolone-resistant strains. The *dfrA1* gene was identified in 20% (4/20) of the trimethoprim-sulfamethoxazole resistant strains and the integrase Class 2 gene was amplified in only 8.2% (5/61) of the isolates. Our findings highlight the emergence of MDR *Salmonella* isolates. A rationalization of antimicrobial use is urgently recommended in both human and veterinary medicine.

## 1. Introduction

Non-typhoid *Salmonella* (NTS) gastroenteritis outbreaks are a major public health problem worldwide [1]. In fact, *Salmonella* serotypes are considered to be the most common zoonotic food-borne pathogens that can be transmitted to humans through food [2]. In 2019, Salmonellosis was the second-most-reported zoonotic disease in the European Union (EU), affecting about 88,000 people [3]. In the USA, the Centers for Disease Control and Prevention (CDC) estimates that NTS causes more food-borne illnesses than any other bacterium [4]. Chicken is a major source of these illnesses [4]. In fact, about 1 in every 25 packages of chicken at the grocery store is contaminated with *Salmonella* [4]. Due to the suspected high correlation between salmonellosis in poultry and the number of human infections, Directive 2003/99/EC of the European Parliament and Council requires that the following five serotypes of *Salmonella* are monitored in poultry flocks: Enteritidis, Typhimurium, Virchow, Hadar and Infantis [5]. In general, contaminated food products (meat, poultry, eggs and dairy) have been reported to be a source of more than 95% NTS gastroenteritis [4]. Symptoms of NTS gastroenteritis include diarrhea, abdominal cramps, fever, vomiting, nausea and headache, which develop within 12 h–72 h and last for 3–7 days [6]. However, up to 5% of the cases may develop invasive extra-intestinal and focal systemic infections requiring effective intravenous (IV) antibiotic therapy or result in death [7]. As a result of extensive and random use of antibiotics in human and veterinary medicine, there has been an alarming increase in the reports that express the spreading of multidrug-resistant (MDR) *Salmonella* strains [8]. Thus, the World Health Organization has defined *Salmonella* as a “priority pathogen” and aims to guide and promote research and development into new antibiotics for its treatment [9]. In fact, in children, the elderly and immune-compromised persons, NTS digestive infections may lead to sepsis and meningitis, and even death, associated with therapeutic failure [4]. Multidrug-resistant (MDR) *Salmonella* strains are an emergent public health worldwide warning [2]. In many African countries, MDR *Salmonella* strains exhibiting resistance to ciprofloxacin have been reported in several studies [10,11,12]. Since 2002 in Europe, the emergence of ciprofloxacin-resistant *Salmonella* isolates has been reported among travelers returning from northeast and eastern Africa [13]. In addition, ampicillin, chloramphenicol and trimethoprim/sulfamethoxazole (SXT) are no longer used as primary antimicrobials because of the high resistance rates of NTS to these agents [14]. Third-generation cephalosporin and fluoroquinolone are recommended as first-line antimicrobials in human use; however, there have been several reports of resistance [15,16].

In Tunisia, data are still lacking about MDR *Salmonella* strains associated with gastroenteritis. The present study aimed to determine the serotype occurrence, virulence factors and antimicrobial resistance patterns of *Salmonella* strains isolated from hospitalized patients with diarrhea/gastroenteritis in Great Tunisia during the last 11 years (2010–2020).

## 2. Results

### 2.1. Occurrence of Salmonella serotypes

Seven serotypes of *Salmonella* were identified in this study; *Salmonella* Enteritidis (27.9%; 17/61), *S*. Typhimurium (26.2%; 16/61) and *S*. Kentucky (24.6%; 15/61) were the most predominant. However, *S*. Anatum (8.2%; 5/61), *S.* Infantis (4.9%; 3/61), *S*. Muenster (4.9%; 3/61) and *S.* Mbandaka (3.3%; 2/61) were rarely found.

The subdivision of the strains according to the years of isolation revealed that from 2010 to 2015, it was only the three predominant serovars (*S.* Enteritidis, *S*. Typhimurium and *S*. Kentucky) that were often identified, but from 2016 new serovars appeared, represented in particular by *S*. Anatum, *S.* Infantis and *S*. Muenster.

### 2.2. Occurrence of Salmonella virulotypes

Analyzed *Salmonella* strains showed five different virulence factor profiles, namely *invA-mgtC-sirA-gipA-pagK* (32.8%; 20/61), *invA-mgtC-sirA-gipA-pagK-Hli* (27.9%; 17/61), *invA-mgtC-sirA-pagK-Hli* (19.7%; 12/61), *invA-mgtC-sirA-pagK* (14.7%; 9/61) and *invA-mgtC-sirA* (4.9%; 3/61). All *Salmonella* strains (61) were positive for the genes *invA* (host cell invasion), *mgtC* (intracellular survival) *and sirA* (control enteropathogenic functions), and were negative for the virulence genes *spvC*, *trhH*, *SEN1417*, *sipA*, *sipD* and *sopD* (Table 1).

### 2.3. Antimicrobial Susceptibility Testing

**Figure 1:**
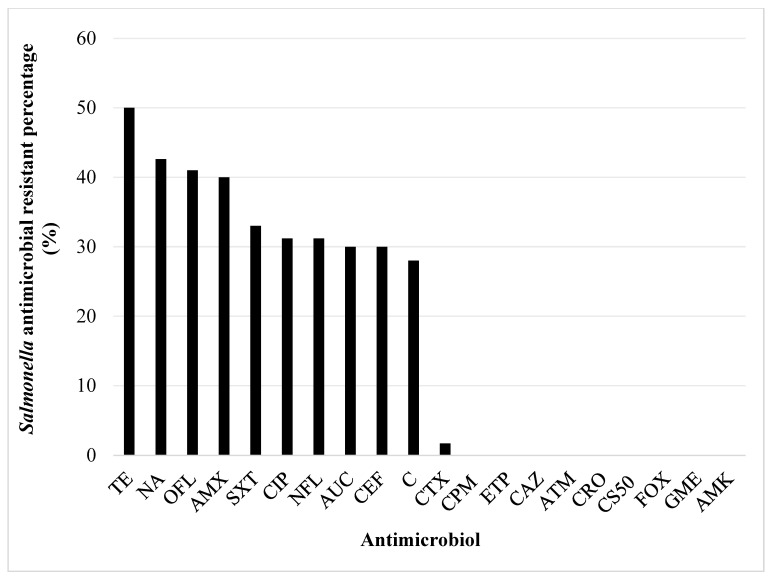
Antimicrobial-resistant percentages in *Salmonella* strains isolated from hospitalized patients with gastroenteritis in Great Tunisia between 2010 and 2020. AMX: Amoxicillin, AUC: Amoxicillin + Clavulanic acid, CEF: Cefalotin, FOX: Cefoxitin, CAZ: Ceftazidim, CTX: Cefotaxim, CRO: Ceftriaxon, CPM: Cefepim, ATM: Aztreonam, ETP: Ertapenem, GME: Gentamicin, CS50: Colistin, NA: Nalidixic acid, OFL: Ofloxacin, CIP: Ciprofloxacin, NFL: Norfloxacin, C: Chloramphenicol, TE: Tetracycline, SXT: Trimethoprim-Sulfamethoxazole and AMK: Amikacin.

Critical resistance rates were detected for tetracycline (50%; 30/61), nalidixic acid (42.6%; 26/61), amoxicillin (40%; 24/61), fluoroquinolones (ofloxacin (41%; 25/61), norfloxacin (31.2%; 19/61), ciprofloxacin (31.2%; 19/61)), trimethoprim-sulfamethoxazole (33%; 20/61), amoxicillin + clavulanic acid (30%; 18/61) and cefalotin (30%; 18/61). However, resistances to ertapenem, ceftazidim, aztreonam, colistin, gentamicin and amikacin (0%; 0/61) were significantly lower than the other antibiotics (*p* < 0.05) (Table 2, Figure 1).

**Figure 2:**
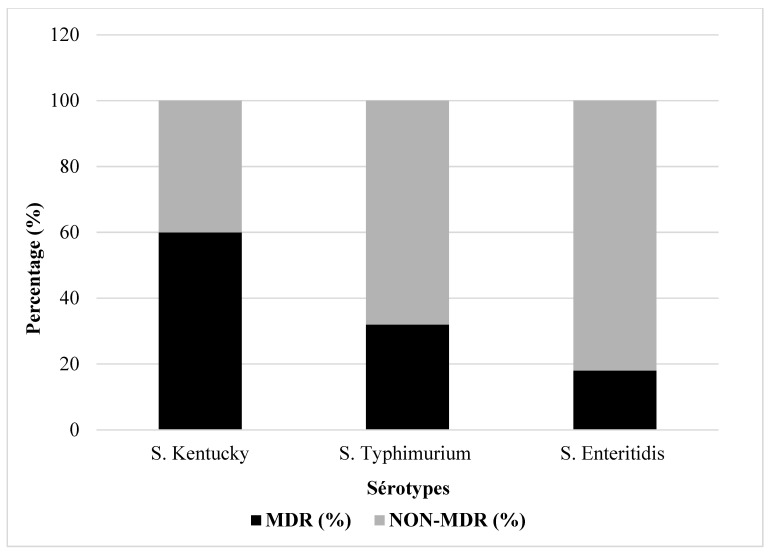
Proportion of MDR *Salmonella* strains according to predominant serotypes.

In addition, 40% (24/61) of the isolated strains were susceptible to all antibiotics used and 33% (20/61) were multidrug-resistant (MDR). Moreover, 80% (12/15) and 60% (9/15) of *S*. Kentucky isolates were identified as fluoroquinolone-resistant and MDR strains, respectively (Table 2, Figure 2).

Moreover, the subdivision of the strains according to the years of isolation revealed the presence of a significant increase in the resistance to tetracycline. Indeed, the tetracycline resistance rate increased from 39% (9/23) to 55% (21/38) during the period from 2017 to 2020. However, the resistance rate to nalidixic acid decreased significantly from 60% (14/23) during the period from 2010 to 2016 to 31% (12/38) during the period from 2017 to 2020. For the other antibiotics, no significant differences in resistance rates were recorded.

### 2.4. Prevalence of Antimicrobial Resistance Genes

Beyond the phenotypic determination, we tested the presence of antimicrobial resistance genes that could have been mediators of antimicrobial resistance in *Salmonella* spp. The *qnrB* gene was amplified in 63.2% (12/19) of the fluoroquinolone-resistant strains. The *dfrA1* gene was identified in 20% (4/20) of the trimethoprim-sulfamethoxazole resistant strains and the integrase Class 2 gene was amplified in only 8.2% (5/61) of the isolates.

## 3. Discussion

In the present study, seven *Salmonella* serotypes were identified, namely *S.* Enteritidis, *S.* Typhimurium, *S*. Kentucky, *S*. Anatum, *S.* Infantis, *S.* Muenster and *S.* Mbandaka. Three serotypes (*S.* Enteritidis, *S.* Typhimurium and *S*. Kentucky) were significantly predominant (*p* < 0.05). Our results showed slight similarities with other studies carried out in other countries such as Greece, Ethiopia, Saudi Arabia, India, China and Korea. Indeed, in all of these studies *S*. Enteritidis and *S*. Typhimurium were the serovars most implicated in NTS gastroenteritis outbreaks [17,18,19,20,21,22,23]. Moreover, *S*. Enteritidis and *S.* Typhimurium are known to be closely associated with broiler flocks, and human infection is generally believed to be derived from poultry and poultry products, including eggs [23,24]. In fact, poultry products are the sources of most NTS food-borne infection cases [2]. In the United States of America, 29% of *Salmonella* strains causing food-borne illnesses were isolated in poultry meat [4].

On the other hand, the high prevalence rate of *S*. Kentucky (24.6%; 15/61) revealed by the current study represents a real threat to human health since it is often associated with multidrug resistance to several antibiotics families, as indicated by Turki et al. (2012) [25]. The increased development in the international food trade and the transport of animals from one country to another are factors favoring the rapid dissemination of S. Kentucky in different regions of the world [26]. The global spread of S. Kentucky, especially in Europe, Africa and Asia, has been illustrated by the isolation of a particular epidemic clone (S. Kentucky ST198-X1) in different reservoirs of livestock, especially in poultry, chicken and turkey farms. For this purpose, poultry are considered the main potential vectors of human infection [26,27].

In this investigation, virulence gene screening classified studied strains into five virulotypes of which 100% were positive for *invA*, *mgtC* and *sirA* and negative for the other virulence genes investigated (*spvC*, *trhH, SEN1417*, *sipA*, *sipD* and *sopD*). These findings are comparable to the results reported by Capuano et al. (2013) who studied 114 *Salmonella* strains of human (71/114) and food (43/114) origin. These strains were screened for 12 virulence factors (*gipA*, *gtgB*, *sopE*, *sspH1*, *sspH2*, *sodC1*, *gtgE*, *spvC*, *pefA*, *mig5*, *rck* and *srgA*) and fifty-nine different virulence profiles were identified with the highest homology related to the presence of prophages (*gipA*, *gtgB*, *sopE*, *sspH1*, *sspH2*, *sodC1* and *gtgE*), while plasmid genes (*spvC*, *pefA*, *mig5*, *rck* and *srgA*) were less detected [8]. Nevertheless, Borah et al. (2021) reported that the virulence genes *invA*, *sipA*, *sipB*, *sipC*, *stn* and *T2544* were identified in all *Salmonella* strains (88). These strains had different origins; they were isolated in humans and several animal species. The *sopB*, *sopE*, *pefA*, *sefC* and *fepA* genes were present in 2/3 of the strains of human and animal origin. The *sefC* gene was present in half of the isolated strains. The multitude of the virulence genes of *Salmonella* highlights the importance of continuous monitoring [28]. In addition, our results revealed that 33% (20/61) of *Salmonella* strains were multi-resistant to antimicrobials considered of great importance for human and animal health. Moreover, 80% (12/15) and 60% (9/15) of *S*. Kentucky isolates were identified as fluoroquinolone-resistant and MDR strains, respectively. This serotype, commonly isolated from poultry carcasses, was reported as being MDR too [29]. In fact, according to the current study, critical resistance rates (>40%) were detected for tetracycline, nalidixic acid, fluoroquinolones, amoxicillin and trimethoprim-sulfamethoxazole. The spread of *Salmonella* with antimicrobial resistance is principally promoted by the use of antibiotics in animal feed to promote the growth of food animals, and in veterinary medicine to treat bacterial infections in those animals [30]. This poses a high risk of zoonotic disease with the transmission of MDR *Salmonella* strains from animals to humans via the ingestion of food or water contaminated with the animals’ feces, direct contact or the consumption of infected food animals [31].

Moreover, *qnrB* and *dfrA1* genes were amplified in 63.2% (12/19) and 20% (4/20) of the fluoroquinolones and trimethoprim-sulfamethoxazole resistant strains, respectively. In the present study, phenotypic resistance is not covered by the occurrence of genes because resistance to an antimicrobial can be linked to different genes, but we cannot test all of these genes. In fact, antibiotic-resistant genes in *Salmonella* spp. induce resistance against common antibiotics. *Salmonella* spp. could obtain resistance to antibiotics through various mechanisms, such as chromosomal mutations, resistance transmission through genetic materials (mostly by plasmids), reduction in cell wall permeability to antibiotics, enzymatic inactivation of antibiotics, efflux pump mechanism for taking out antibiotics and the altered target position of antibiotics [32]. Our findings do not corroborate with the results of Borah et al. (2021), indicating high rates of resistance (more than 60%) to ampicillin, tetracycline and cefotaxime and moderate resistance rates (almost 10%) to ceftriaxon, chloramphenicol, ciprofloxacin and cefepim in 88 *Salmonella* strains. *bla_TEM_*, *tetA* and *dfrA12* resistance genes were the most prevalent; they were isolated in strains of different origin [28]. Moreover, according to the current study, the subdivision of the strains according to the years of isolation revealed the presence of a significant increase in the resistance to tetracycline. Indeed, the tetracycline resistance rate increased from 39% (9/23) to 55% (21/38) during the period from 2017 to 2020. However, the resistance rate to nalidixic acid decreased significantly from 60% (14/23) during the period from 2010 to 2016 to 31% (12/38) during the period from 2017 to 2020. For the other antibiotics, no significant difference in resistance rates was recorded.

Then, according to the present study and other reports [33,34,35], extended-spectrum cephalosporines (especially ceftriaxon), aztreonam and ertapenem, should be suggested as the drugs of choice for treating patients with serious NTS infections. In addition, clinicians should be vigilant toward the emergence of MDR *Salmonella* strains with decreased susceptibility to extended-spectrum cephalosporines. Nevertheless, the prevention of NTS gastroenteritis requires the vigorous application of hygienic good practices regarding hands, food and healthcare management which is firmly the most efficient strategy to deal with infections and limit the spread of antimicrobial resistance [33].

## 4. Materials and Methods

### 4.1. Sample Collection and Salmonella Identification

A total of 61 *Salmonella* strains were isolated from hospitalized patients with gastroenteritis at a university hospital in Great Tunisia between 2010 and 2020. The isolation of *Salmonella* from the selective enrichment SX2 broth (bioMérieux SA, Lyon, France) previously inoculated by a clinical specimen and incubated at 37 °C for 24 h, on selective agar (XLD and SS) (Biokar Diagnostics, Beauvais, France), was conducted following the reference technique indicated in REMIC, 2018 [36]. The identification and confirmation of *Salmonella* strains were performed via the urease test followed by the API 20E system test (bioMérieux SA, Lyon, France) according to the reference technique indicated in REMIC, 2018 [36].

### 4.2. Salmonella Strain Serotyping

Serotyping was carried out on *Salmonella* isolates according to the White–Kauffmann-Le Minor scheme [37]. Slide agglutination tests were performed with specific immune sera against *O*, *H* and *Vi Salmonella* antigens (BioRad, Marne-La-Coquette, France).

### 4.3. Molecular Study

*Salmonella* genomic DNA extraction was performed from the selective enrichment SX2 broth (bioMérieux SA, Lyon, France) according to the manufacturer’s guidelines using the *ONE-4-ALL GENOMIC DNA Mini-Preps Kit* (Bio Basic, Markham, Canada). *Salmonella* genomic DNA was stored at −20 °C until it was used.

The specific primers (F-5’ACCACGCTCTTTCGTCTGG3’ and R-5GAACTGACTACGTAGACGCTC3’) [11] were used for *Salmonella* molecular confirmation and all isolates were tested for 12 invasion and virulence genes (Table 3).

The PCR was conducted in a total reaction volume of 25 µL consisting of 1 U Taq Polymerase, 1.5 mM MgCl_2_, 0.1 mM dNTPs, 1 µM forward and reverse primer (Bio Basic, Markham, ON, Canada), 1x PCR buffer (5 mM KCl Tris-HCl, pH 8.5) and 1 µL of DNA. The first denaturation was conducted at 95 °C for 10 min followed by 35 cycles (denaturation for 1 min at 95 °C, annealing for 1 min at different temperatures according to the target gene and elongation for 1 min at 72 °C) and the last elongation was performed for 10 min at 72 °C. All of the PCR reactions were performed in an Esco Swift Max Pro thermocycler (Horsham PA, USA).

The primers (Bio Basic, Markham, Canada) used for detecting virulence and the antimicrobial resistance genes of *Salmonella* are listed in Table 3 and Table 4.

Gene amplicons were visualized in 1.5% agarose gel stained with ethidium bromide and photographed under UV transilluminate. Positive and negative controls represented by *Salmonella* strains isolated in a previous study [27,52] and distilled sterile water were added in each PCR run, respectively.

### 4.4. Antimicrobial Susceptibility Testing and Identification of Antimicrobial Resistance Genes

The antimicrobial susceptibility tests were performed using the agar disc diffusion method on Mueller–Hinton agar (Bio-Rad, Marne-La-Coquette, France) plates. *Salmonella* isolated strains were examined for their susceptibility to 20 antibiotic discs (Bio-Rad, Marne-La-Coquette, France). The inhibition zone was measured to survey the resistance or the susceptibility according to the interpretation criteria established by the Antibiogram *Committee of the French Society* for *Microbiology* [53]. The Colispot test was performed for detecting colistin resistance. The resistant *Salmonella* strains were revealed by the absence of an inhibition zone after an application of a single drop of 8 mg/L colistin solution on previously inoculated Mueller–Hinton agar [54]. The screening of resistance genes beta-lactams (*bla_TEM_*, *bla_NDM1_* and *bla_CTX-M_*), fluoroquinolones (*qnrB*), tetracycline (*tet(A)* and *tet(B)*), trimethoprim (*dfrA1*) and colistin (*mcr-1 to mcr-5*) indicated in Table 4 [40,41,42,43,44,45,46,47] was carried out by performing PCRs as described above. Class 1 and Class 2 integrons were screened in all of the *Salmonella* isolated strains using primers described by Mazel et al. (2000) [51].

### 4.5. Statistical Analysis

*Salmonella* serotypes, virulotypes and antimicrobial resistance percentages were compared using Epi Info 6 with the Mantel Haenszel chi-square test. Observed differences were considered significant when the *p* value was lower than 0.05.

## 5. Conclusions

The current study reported the predominance of three serovars represented by *S.* Enteritidis, *S*. Typhimurium and *S.* Kentucky in isolated *Salmonella* strains from hospitalized patients with gastroenteritis in Great Tunisia between 2010 and 2020. In addition, more than half of the *S*. Kentucky isolates were identified as being fluoroquinolone-resistant and MDR strains. Moreover, high resistance rates were detected for tetracycline, nalidixic acid, fluoroquinolones, amoxicillin and trimethoprim-sulfamethoxazole. However, resistance rates to cephalosporines 3/4G, ertapenem, aztreonam, colistin, gentamicin and amikacin were usually lower than 5%. Our findings highlight the emergence of multidrug-resistant *Salmonella* isolates in Tunisia with decreased susceptibility to many antimicrobials. To this end, a rationalization of antimicrobial use is urgently recommended in both human and veterinary medicine.

## Figures and Tables

**Table 1:** Serotypes and virulence genes of *Salmonella* isolated in the present study.

Serotypes (%)	Strains	Virulence Genes ^(a)^											
		*invA*	*mgtC*	*sirA*	*gipA*	*pagK*	*hli*	*trhH*	*spvC*	*sipA*	*sipD*	*sopD*	*SEN 1417*
*S.* Enteritidis 27.9% (17/61)	600/20	+	+	+	+	+	-	-	-	-	-	-	-
	645/19	+	+	+	-	+	+	-	-	-	-	-	-
	888/19	+	+	+	+	+	-	-	-	-	-	-	-
	644/18	+	+	+	+	+	-	-	-	-	-	-	-
	697/18	+	+	+	-	+	+	-	-	-	-	-	-
	148/17	+	+	+	+	+	-	-	-	-	-	-	-
	323/17	+	+	+	+	+	-	-	-	-	-	-	-
	1102/17	+	+	+	+	+	-	-	-	-	-	-	-
	1456/16	+	+	+	+	+	-	-	-	-	-	-	-
	984/14	+	+	+	-	+	-	-	-	-	-	-	-
	29/12	+	+	+	+	+	-	-	-	-	-	-	-
	732/12	+	+	+	+	+	-	-	-	-	-	-	-
	735/12	+	+	+	+	+	-	-	-	-	-	-	-
	2157/12	+	+	+	-	+	-	-	-	-	-	-	-
	621/11	+	+	+	+	+	-	-	-	-	-	-	-
	1038/11	+	+	+	+	+	-	-	-	-	-	-	-
	1749/10	+	+	+	+	+	-	-	-	-	-	-	-
*S.* Typhimurium 26.2% (16/61)	17/20	+	+	+	-	+	-	-	-	-	-	-	-
	18/20	+	+	+	+	+	+	-	-	-	-	-	-
	828/19	+	+	+	-	+	+	-	-	-	-	-	-
	1007/18	+	+	+	-	+	+	-	-	-	-	-	-
	1017/18	+	+	+	+	+	+	-	-	-	-	-	-
	211/17	+	+	+	-	+	+	-	-	-	-	-	-
	859/17	+	+	+	+	+	+	-	-	-	-	-	-
	860/17	+	+	+	+	+	+	-	-	-	-	-	-
	861/17	+	+	+	+	+	+	-	-	-	-	-	-
	862/17	+	+	+	+	+	+	-	-	-	-	-	-
	863/17	+	+	+	+	+	+	-	-	-	-	-	-
	880/17	+	+	+	-	+	+	-	-	-	-	-	-
	881/17	+	+	+	+	+	+	-	-	-	-	-	-
	1103/17	+	+	+	-	+	+	-	-	-	-	-	-
	1523/12	+	+	+	+	+	+	-	-	-	-	-	-
	2156/12	+	+	+	+	+	+	-	-	-	-	-	-
*S.* Kentucky 24.6% (15/61)	319/20	+	+	+	+	+	+	-	-	-	-	-	-
	981/19	+	+	+	+	+	+	-	-	-	-	-	-
	69/18	+	+	+	-	+	+	-	-	-	-	-	-
	322/17	+	+	+	+	+	-	-	-	-	-	-	-
	1212/17	+	+	+	+	+	-	-	-	-	-	-	-
	1213/17	+	+	+	+	+	+	-	-	-	-	-	-
	1278/17	+	+	+	+	+	+	-	-	-	-	-	-
	390/16	+	+	+	-	+	+	-	-	-	-	-	-
	1457/16	+	+	+	+	+	+	-	-	-	-	-	-
	1821/15	+	+	+	+	+	+	-	-	-	-	-	-
	774/13	+	+	+	+	+	+	-	-	-	-	-	-
	1114/12	+	+	+	-	+	+	-	-	-	-	-	-
	1119/12	+	+	+	-	+	+	-	-	-	-	-	-
	171/11	+	+	+	-	+	+	-	-	-	-	-	-
	622/11	+	+	+	-	-	-	-	-	-	-	-	-
*S.* Anatum 8.2% (5/61)	333/20	+	+	+	-	+	-	-	-	-	-	-	-
	598/18	+	+	+	-	+	-	-	-	-	-	-	-
	1268/17	+	+	+	+	+	-	-	-	-	-	-	-
	391/16	+	+	+	+	+	-	-	-	-	-	-	-
	1750/10	+	+	+	-	-	-	-	-	-	-	-	-
*S.* Infantis 4.9% (3/61)	17/18	+	+	+	-	+	-	-	-	-	-	-	-
	684/18	+	+	+	-	+	-	-	-	-	-	-	-
	1006/18	+	+	+	-	+	-	-	-	-	-	-	-
*S.* Muenster 4.9% (3/61)	1210/17	+	+	+	-	+	-	-	-	-	-	-	-
	1211/17	+	+	+	+	+	-	-	-	-	-	-	-
	1093/16	+	+	+	-	-	-	-	-	-	-	-	-
*S.* Mbandaka 3.3% (2/61)	226/18	+	+	+	+	+	-	-	-	-	-	-	-
	804/15	+	+	+	+	+	-	-	-	-	-	-	-

^(a)^ +: present; -: absent.

**Table 2:** Serotypes and antimicrobial resistance profiles of *Salmonella* isolated in the present study.

Serotypes (%)	Strains	Antibiotic Resistance Profiles ^(a)^																			
		AMX	AUC	CEF	CAZ	CTX	ATM	CRO	CPM	FOX	ETP	NA	NFL	CIP	OFL	TE	SXT	C	CS50	GME	AMK
*S.* Enteritidis 27.9% (17/61)	600/20																				
	645/19																				
	888/19																				
	644/18																				
	697/18 *																				
	148/17 *																				
	323/17																				
	1102/17																				
	1456/16																				
	984/14 *																				
	29/12																				
	732/12																				
	735/12																				
	2157/12																				
	621/11																				
	1038/11																				
	1749/10																				
*S.* Typhimurium 26.2% (16/61)	17/20																				
	18/20 *																				
	828/19 *																				
	1007/18																				
	1017/18																				
	211/17																				
	859/17 *																				
	860/17																				
	861/17																				
	862/17																				
	863/17																				
	880/17																				
	881/17 *																				
	1103/17																				
	1523/12 *																				
	2156/12																				
*S.* Kentucky 24.6% (15/61)	319/20																				
	981/19 *																				
	69/18																				
	322/17 *																				
	1212/17 *																				
	1213/17 *																				
	1278/17 *																				
	390/16																				
	1457/16 *																				
	1821/15 *																				
	774/13 *																				
	1114/12 *																				
	1119/12																				
	171/11																				
	622/11																				
*S.* Anatum 8.2% (5/61)	333/20																				
	598/18																				
	1268/17																				
	391/16																				
	1750/10																				
*S.* Infantis 4.9% (3/61)	17/18																				
	684/18																				
	1006/18 *																				
*S.* Muenster 4.9% (3/61)	1210/17 *																				
	1211/17																				
	1093/16																				
*S.* Mbandaka 3.3% (2/61)	226/18																				
	804/15 *																				

^(a)^ AMX: Amoxicillin, AUC: Amoxicillin + Clavulanic acid, CEF: Cefalotin, FOX: Cefoxitin, CAZ: Ceftazidim, CTX: Cefotaxim, CRO: Ceftriaxon, CPM: Cefepim, ATM: Aztreonam, ETP: Ertapenem, GME: Gentamicin, CS50: Colistin, NA: Nalidixic acid, OFL: Ofloxacin, CIP: Ciprofloxacin, NFL: Norfloxacin, C: Chloramphenicol, TE: Tetracycline, SXT: Trimethoprim-Sulfamethoxazole and AMK: Amikacin. Susceptible 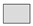 Resistant 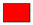. * MDR strains.

**Table 3:** Primers of *Salmonella* for virulence genes targeted in the present study.

Gene	Function	Primer Sequence (5′ to 3′)	Product Size (bp)	Annealing Temperature (°C)	Reference
*SEN1417*	Intracellular survival	F: GATCGCTGGCTGGTC	670	58	[38]
		R: CTGACCGTAATGGCGA			
*sipA*	Host cell invasion	F: ATGGTTACAAGTGTAAGGACTCAG	2055	53	[39]
		R: ACGCTGCATGTGCAAGCCATC			
*sipD*	Host cell invasion	F: ATGCTTAATATTCAAAATTATTCCG	1029	53	[39]
		R: TCCTTGCAGGAAGCTTTTG			
*sopD*	Host cell invasion	F: GAGCTCACGACCATTTGCGGCG	1291	59	[40]
		R: GAGCTCCGAGACACGCTTCTTCG			
*gipA*	Growth or survival in a Peyer’s patch	F: ACGACTGAGCAGGCTGAG	518	58	[41]
		R: TTGGAAATGGTGACGGTAGAC			
*mgtC*	Intracellular survival	F: TGACTATCAATGCTCCAGTGAAT	677	58	[41]
		R: ATTTACTGGCCGCTATGCTGTTG			
*trhH*	Code for the putative F pilus assembly protein	F: AACTGGTGCCGTTGTCATTG	418	53	[41]
		R: GATGGTCTGTGCTTGCTGAG			
*spvC*	Multiplication in host cell	F: CTCCTTGCACAACCAAATGCG	570	53	[41]
		R: TGTCTCTGCATTTCACCACCATC			
*sirA*	Control enteropathogenic virulence functions	F: TGCGCCTGGTGACAAAACTG	313	55	[41]
		R: ACTGACTTCCCAGGCTACAGCA			
*pagK*	Biofilm formation	F: ACCATCTTCACTATATTCTGCTC	151	60	[41]
		R: ACCTCTACACATTTTAAACCAATC			
*invA*	Host cell invasion	F: GTGAAATTATCGCCACGTTCGGGCAA	284	64	[42]
		R: TCATCGCACCGTCAAAGGAACC			
*Hli*	Control of phase change and motility	F: AGCCTCGGCTACTGGTCTTG	173	55	[43]
		R: CCGCAGCAAGAGTCACCTCA			

F: forward primer; R: reverse primer.

**Table 4:** Primers of *Salmonella* for antibiotic resistance genes targeted in the present study.

Gene	Primer Sequence (5′ to 3′)	Product Size (bp)	Annealing Temperature (°C)	Reference
*bla_TEM_*	F: ATCAGCAATAAACCAGC	516	54	[44]
	R: CCCCGAAGAACGTTTTC			
*bla_CTX-M_*	F: ATGTGCAGYACCAGTAARGTKATGGC	592	58	[45]
	R: TGGGTRAARTARGTSACCAGAAYSAGCGG			
*bla_NDM1_*	F: CTGAGCACCGCATTAGCC	621	52	[46]
	R: GGGCCGTATGAGTGATTGC			
*tetA*	F: GGTTCACTCGAACGACGTCA	577	55	[47]
	R: CTGTCCGACAAGTTGCATGA			
*tetB*	F: CCTCAGCTTCTCAACGCGTG	634	55	[47]
	R: GCACCTTGCTGATGACTCTT			
*dfrA1*	F: GGAGTGCCAAAGGTGAACAGC	367	55	[48]
	R: GAGGCGAAGTCTTGGGTAAAAAC			
*qnrB*	F: GATCGTGAAAGCCAGAAAGG	469	53	[49]
	R: ACGATGCCTGGTAGTTGTCC			
*mcr-1*	F: AGTCCGTTTGTTCTTGTGGC	320	58	[50]
	R: AGATCCTTGGTCTCGGCTTG			
*mcr-2*	F: CAAGTGTGTTGGTCGCAGTT	715	58	[50]
	R: TCTAGCCCGACAAGCATACC			
*mcr-3*	F: AAATAAAAATTGTTCCGCTTATG	929	58	[50]
	R: AATGGAGATCCCCGTTTTT			
*mcr-4*	F: TCACTTTCATCACTGCGTTG	1116	58	[50]
	R: TTGGTCCATGACTACCAATG			
*mcr-5*	F: ATGCGGTTGTCTGCATTTATC	1644	58	[50]
	R: TCATTGTGGTTGTCCTTTTCTG			
*int1*	F: GGGTCAAGGATCTGGATTTCG	483	62	[51]
	R: ACATGGGTGTAAATCATCGTC			
*int2*	F: CACGGATATGCGACAAAAAGGT	233	62	[51]
	R: GTAGCAAACGAGTGACGAAATG			

F: forward primer; R: reverse primer.

## Data Availability

The study did not report any data.
